# Rutin Improved the Meat Quality and Hepatointestinal Health of Nile Tilapia (*Oreochromis niloticus*) After High‐Level Fava Bean Feeding

**DOI:** 10.1155/anu/3152165

**Published:** 2026-01-11

**Authors:** Ke Cheng, Xinyao Zhang, Lixue Dong, Di Peng, Yangyang Liu, Dexing Zhu, Zhongbao Guo, Yongju Luo, Apeng Liu, Juan Tian, Hua Wen, Mingdian Liu, Ming Jiang

**Affiliations:** ^1^ Yangtze River Fisheries Research Institute, Chinese Academy of Fishery Sciences, Wuhan, 430223, China, cafs.ac.cn; ^2^ Fisheries Research and Extension Center of Huizhou, Huizhou, 516000, Guangdong, China; ^3^ Guangxi Academy of Fishery Sciences, Nanning, 530021, Guangxi, China; ^4^ Shenzhen Aohua Group Co., Ltd., Shenzhen, 518054, Guangdong, China

**Keywords:** fava bean, intestinal microbe, muscle, Nile tilapia, rutin

## Abstract

A high‐level fava beans diet has been proven to enhance the texture characteristics of tilapia muscle, yet it can also induce hepatointestinal injury. Rutin, as a nutritional additive, has antioxidant and immune‐boosting effects. This study explored the regulatory effect of 150 and 300 mg/kg rutin on the muscle characteristics and hepatointestinal health of tilapia after being fed with a 60% fava beans diet. Two hundred and forty tilapias (average weight: 371.50 ± 9.29 g) were evenly divided into four groups and fed with the diet of groups C0 (0% fava bean), R0 (60% fava bean), R1 (60% fava bean + 150 mg/kg rutin), and R2 (60% fava bean + 300 mg/kg rutin) for 10 weeks, respectively. The results indicated that the growth performance in R1 and R2 showed no significant changes compared to R0, while muscle hardness, gumminess, and resilience increased, along with an elevation in muscle crude protein deposition. The addition of 150 and 300 mg/kg rutin enhanced the antioxidant capacity (AOC) of the muscles, liver, and intestines, alleviated liver damage by regulating liver lipid metabolism compared with R0. Dietary supplementation with 150 mg/kg rutin improved the composition of intestinal microbiota, significantly upregulating the abundance of beneficial bacteria *Muribaculaceae* unclassified and reduced the abundance of harmful bacteria *Rhodobacteraceae*, *Chloroplast_unclassified*, and *Rothia*. In summary, rutin can effectively function as a nutritional supplement to alleviate liver and intestinal damage caused by 60% fava bean feed, while also improving muscle texture simultaneously. This study provides a crucial theoretical basis for optimizing crispy tilapia aquaculture through rutin supplementation, promoting innovation in aquatic feed formulations.

## 1. Introduction

Tilapia (*Oreochromis niloticus*) is one of the most important farmed fish species in the world due to its high yield, strong adaptability, and rich nutritional value. Crispy is an innovative aquaculture technique that enhances the muscle hardness and chewiness of fish by incorporating a specific proportion of fava beans (*Vicia faba* L.) into their diet, thereby addressing the relatively soft texture commonly associated with traditionally farmed tilapia. Currently, the application of crispy technology in the aquaculture of grass carp (*Ctenopharyngodon idellus*) and tilapia has been preliminary researched and implemented [1, 2]. This has notably enhanced the economic returns of the aquaculture industry. As for tilapia, studies have shown that the addition of fava beans increases the muscle fiber density [3], alters the composition of flavor amino acids, and thereby enhances the special texture of the muscle [3, 4]. However, previous studies conducted by our laboratory have demonstrated that dietary supplementation of 50%–70% fava beans can also cause significant liver damage [5]. In addition, Li et al. [6] pointed that feeding fava beans significantly reduced the growth performance and significantly increased liver and serum transaminase levels of tilapia. The evidence presented suggests that fava bean‐rich diets enhance fish meat quality, though this improvement frequently coincides with health deterioration in farmed fish. Therefore, investigating strategies to mitigate liver and intestine damage during crispy aquaculture holds substantial theoretical and practical significance.

Rutin, also known as vitamin P, has the molecular formula of C_27_H_30_O_16_ and is a flavonoid substance extracted from plants. Extensive research has demonstrated the therapeutic potential of rutin in safeguarding hepatic and gastrointestinal functions across animal models. In mammals, rutin exhibits anti‐inflammatory properties by counteracting DSS–induced mucosal damage in murine colonic tissues and suppressing key pro‐inflammatory cytokines including IL‐1β and IL‐6 [7]. It could inhibit liver inflammation induced by CCl4 through the inhibition of the TLR4/MyD88/NF‐κB pathway [8]. In aquatic animals, Liu et al. [9] reported that 100 mg/kg rutin can enhance the growth performance and intestinal health of juvenile yellow catfish (*Pelteobagrus fulvidraco*). Nazeri et al. [10] confirmed that rutin can alleviate liver damage in rainbow trout (*Oncorhynchus mykiss*) exposure to Oxytetracycline by enhancing antioxidant capacity (AOC). In addition, Xu et al. [11] found through metabolomics analysis that rutin could improve the muscle growth and meat quality of grass carp. Therefore, this study hypothesized that moderate amounts of rutin can alleviate hepatointestinal damage induced by dietary fava beans, and may further improve the texture characteristics of tilapia muscle.

Therefore, a 10‐week feeding trial was carried out to assess the effects of rutin on the growth performance, muscle texture characteristics, as well as hepatointestinal injury of the tilapia fed with 150 and 300 mg/kg rutin based on 60% fava bean supplementation. This study can provide a theoretical foundation for optimizing the crisping culture technology of tilapia and promote the innovation and development of aquatic feed.

## 2. Materials and Methods

### 2.1. Animal Ethics

All experimental operations comply with the requirements of the Laboratory Animal Centre of the Yangtze River Fisheries Research Institute, Chinese Academy of Fishery Sciences (License Number: YFI2022JM06).

### 2.2. Feeding Trial

This study consisted of four types of extruded experimental feeds with a diameter ranging from 6.80 to 7.00 mm: the negative control diet with 0% fava bean addition (C0), the positive control diet with 60% fava bean addition (R0), the treatment diet with 60% fava bean and 150 mg/kg rutin addition (R1), and the treatment diet with 60% fava bean and 300 mg/kg rutin addition (R2). The feed formula and nutritional compositions were shown in Table 1. The experimental Nile tilapia was purchased from a tilapia farm located on Nanhua 1st Road, Jinwan District, Zhuhai City, Guangdong Province, China, and placed in pond net cages (2.0 m × 1.0 m × 1.5 m) for temporary cultivation for 2 weeks. During the temporary feeding period, a mixed feed of C0 and R0 (mixed 1:1 by weight) was provided. After temporary cultivation, 240 tilapia with consistent specifications (initial average body weight: 371.50 ± 9.29 g, *n* = 20) were randomly selected and placed in a net cage for every 20 fish (aquaculture density: 3.093 kg/m^3^). There were four experimental groups with three replicates in each group. The growth trial lasted for 10 weeks, with feeding twice a day at 8 : 00 and 17 : 00, and a feeding amount of 2% of the total weight of the fish. Water quality parameters: the dissolved oxygen in the water was greater than 4.5 mg/L, the ammonia nitrogen was less than 0.5 mg/L, the pH was about 7.5–8.0, and the temperature ranged from 25.0–28.0°C (measured at 0.5 m underwater).

**Table 1:** Formulation and proximate composition of the test diets (as air‐dried basis, g/kg).

Ingredients	C0	R0	R1	R2
Corn gluten meal^a^	160.00	0.00	0.00	0.00
Wheat starch^b^	60.00	30.00	29.85	29.70
Fish meal^c^	20.00	20.00	20.00	20.00
Chicken powder^d^	20.00	20.00	20.00	20.00
Soybean meal^e^	355.00	171.50	171.50	171.50
Rapeseed meal^f^	140.00	70.00	70.00	70.00
Fava bean^g^	0.00	600.00	600.00	600.00
Corn DDGS^h^	155.50	0.00	0.00	0.00
Soybean oil^i^	55.00	55.00	55.00	55.00
Monocalcium phosphate^j^	18.00	18.00	18.00	18.00
Rutin^k^	0.00	0.00	0.15	0.30
Vitamin premix^l^	5.00	5.00	5.00	5.00
L‐ascorbate‐2‐monophosphate	1.00	1.00	1.00	1.00
Mineral premix^m^	5.00	5.00	5.00	5.00
Choline chloride	1.50	1.50	1.50	1.50
Lysine	2.00	0.00	0.00	0.00
DL‐Met	2.00	3.00	3.00	3.00
Total	1000.00	1000.00	1000.00	1000.00
Proximate composition (as fed, g/kg)				
Crude protein	266.00	269.00	260.00	264.00
Crude lipid	49.00	54.00	45.00	44.00
Mositure	88.00	75.00	80.00	99.00
Ash	70.00	69.00	66.00	62.00
Lysine^n^	1.67	1.90	1.94	1.97
Met^n^	0.55	0.44	0.41	0.38

^a^Corn meal: Henan Julong Bioengineering Co., Ltd. Basic nutritional components: crude fat 2.8%, crude protein 8%, moisture 14%, ash 1.2%, crude fiber 2.8%, Ca 0.17%, P 0.41%, DL‐Met 0.16%, Lys 0.24%.

^b^Wheat starch: Yihaijiali (Wuhan) Grain and Oil Industry Co., Ltd.

^c^Fish meal: Origin from Zhoushan, Zhejiang, China.

^d^Chicken powder: Yixiang (Zhejiang) Biotechnology Co., Ltd., Huzhou, Zhejiang, China.

^e^Soybean meal: Cofco (Dongguan) Grain and Oil Industry Co., Ltd., Beijing, China. Basic nutritional components: crude fat 4%, crude protein 46%, moisture 13%, ash 5%, crude fiber 4%, Ca 0.34%, P 0.65%, DL‐Met 0.62%, Lysine 2.71%.

^f^Rapeseed meal: Xinjiang Jianlan Plant protein Co., Ltd., Shihezi, Xinjiang, China. Basic nutritional components: crude fat 2%, crude protein 38.5%, moisture 11%, ash 7.5%, crude fiber 13%, Ca 0.65%, P 1.02%, DL‐Met 0.74%, Lys 1.75%.

^g^Fava bean: Xianning, Hubei, China. Basic nutritional components: crude fat 1.7%, crude protein 27%, moisture 12%, ash 3.4%, crude fiber 8.5%, Ca 0.14%, P 0.42%, DL‐Met 0.19%, Lys 1.65%.

^h^Corn DDGS: corn distillers dried grains with solubles.

^i^Soybean oil: Yihaijiali (Wuhan) Grain and Oil Industry Co., Ltd., Shnaghai, China.

^j^Monocalcium phosphate: as a phosphate source.

^k^Rutin: ≥97% purity, Shanghai yuanye Bio‐Technology Co., Ltd.

^l^Vitamins premix (per kg of dry feed): retinol acetate 5000 IU; cholecalciferol 2000 IU; α‐tocopheryl acetate 60 mg; L‐ascorbyl‐2‐monophosphate‐Mg 120 mg; menadione 5 mg; thiamin hydrochloride 5 mg; riboflavin 20 mg; pyridoxine hydrochloride 10 mg; nicotinic acid 120 mg; calcium pantothenate 10 mg; folic acid 1 mg; biotin 0.1 mg; inositol 400 mg.

^m^Minerals premix (per kg of dry feed): Ca(CH_3_CHOHCOO)_2_ 6540 mg, FeSO_4_ 42.5 mg, MgSO_4_ 1340 mg, NaH_2_PO_4_ 1744 mg, NaCl 870 mg, AlCl_3_ 3 mg, KIO_3_ 2.5 mg, KCl 1500 mg, CuCl_2_ 2 mg, MnSO_4_ 16 mg, CoCl_2_ 20 mg, ZnSO_4_ 60 mg.

^n^indicates the calculated value.

### 2.3. Sample Collection

After feeding trail, the fish were fasted for 24 h, and all fish in each cage were counted and weighed. Six fish were randomly selected from each cage and placed in a plastic bucket containing 40 mg/L MS‐222 (Aladdin, Shanghai, China) for anesthesia to measure the body length and weight. Subsequently, blood samples were collected from the tail vein using a 2 mL sterile syringe. A portion of the blood was placed in a test tube containing anticoagulant and immediately used for blood cell analysis. The remaining portion was left to stand at 4°C for 4 h, then, centrifuged at 960 × *g* for 10 min, and the supernatant was collected and stored in a refrigerator at −80°C until the serum biochemical indicators were detected. After blood collection, the dose of MS‐222 was increased to 100 mg/L to euthanize the experimental fish and then perform dissection. The weights of the visceral masses and liver were weighed, respectively. Following dissection of intestinal tissue from the visceral masses, mid‐intestinal along with liver and muscle tissues were obtained and immersion‐fixed in 4% paraformaldehyde (Labgic, Beijing, China). The remaining intestinal, liver, and muscle samples were flash‐frozen in liquid nitrogen prior to long‐term preservation at −80°C for subsequent detection of physiological and biochemical indicators. Another three tilapia were taken from each cage and anesthetized according to the above method. The bilateral dorsal muscles were collected for the detection of basic nutrient compositions. Another three tilapia were taken and the dorsal muscles at the same position on one side were collected for the detection of texture characteristics. Finally, the intestinal contents of every three fish were mixed into one sample and stored at −80°C until intestinal microbiome detection.

### 2.4. Growth Performance Measurement

The feeding status of tilapia was observed daily. After a 10‐week feeding trial, body weight, body length, visceral masses, and liver weight were measured to calculate the weight gain (WG), specific growth rate (SGR), hepatosomatic index (HSI), viscerosomatic index (VSI), and condition factor (CF) of tilapia.

### 2.5. Detection of Blood Cell Composition and Serum Biochemical Indicators

The whole blood samples were analyzed for blood cell components using the Mindray Veterinary fully automatic blood cell analyzer (Mindray, BC‐2800vet, Shenzhen, China). And the level of alkaline phosphatase (ALP), aspartate aminotransferase (AST), alanine aminotransferase (ALT), total protein (TP), serum albumin (ALB), glucose (GLU), triglyceride (TG), and total cholesterol (T‐CHO) in serum samples were determined in the fully automatic biochemical analyzer (Sysmex, BX‐3010, Japan).

### 2.6. Determination of Nutrient Composition in Muscle

Moisture was detected using oven drying method. The sample was dried at 105°C to a constant weight and the weight loss ratio was calculated. The crude protein was detected using the Kjeldahl method [12] (method 984.13), and the sample was digested with sulfuric acid and analyzed on an automatic nitrogen analyzer. The Soxhlet extraction procedure [12] (method 920.39) was employed to quantify crude fat content. The fat in the sample was extracted with ether, and the solvent was evaporated before weighing. The ash content was detected by high‐temperature burning method [12] (method 942.05), and the sample was burned to constant weight at 550°C. The residual inorganic substances were the ash content.

### 2.7. Analysis of Muscle Texture

The dorsal muscles at the same position on one side of the tilapia were cut into 1.0 cm × 1.0 cm × 1.0 cm cubes, and three samples were taken from each fish. Texture characteristics analysis was conducted using a TVT300XP texture analyzer (TexVol, Sweden), with testing parameters adopted from prior methodology [13].

### 2.8. Histomorphological Observation

The muscle, liver and middle intestine samples were subjected to dehydration, embedding and slicing, and then, the slices were subjected to hematoxylin–eosin (H.E.) staining to analyze histopathological alterations. Among them, liver slices also need to undergo Oil red O and Masson staining respectively to determine the degree of lipid deposition and collagen fibrosis in the liver. The histotechnical procedures including tissue sectioning and staining were conducted by Wuhan Servicebio Technology Co., Ltd., with subsequent microscopic analysis performed using optical microscopy. Each group consisted of three repeated slices, from which three to five fields of view were selected and photographed at 200x magnification. Image analysis was conducted with Image J (v1.53). For H.E. stained slices, quantitative indicators of the intestine comprised the villi number, length, width, and muscularis thickness; while for muscle slices, the indicators included the long diameter, short diameter, cross‐sectional area, and density of muscle fibers. Statistical analysis was performed to quantify the proportional areas of lipid droplets and collagen fibers in liver slices stained with both Oil Red O and Masson techniques.

### 2.9. Determination of Tissue Physiological and Biochemical Indicators

The obtained muscle, liver, and middle intestine samples were mixed with an appropriate amount of precooled PBS to create a 10% tissue suspension, which was then centrifuged at 4°C and 4000 rpm for 10 min to obtain the supernatant. The obtained supernatant was used to detect protein concentration (BCA method), malondialdehyde (MDA), total AOC (T‐AOC), catalase (CAT), superoxide dismutase (SOD), lipase (LPS), trypsin, hydroxyproline, TGs, and T‐CHO. All indicators was performed in accordance with the instructions of the test kit of Nanjing Jiangcheng Bioengineering Institute (Nanjing, China) and was determined using an microplate reader (MinDRay BS‐400, Shenzhen, China).

### 2.10. Detection of the Intestinal Microbiome

The obtained intestinal contents underwent instestinal microbiome analysis, following this brief procedure: Initially, genomic DNA was extracted from the samples, primers were designed and synthesized, and the target sequencing fragments were amplified by PCR. Subsequently, the PCR products were quantified and normalized, libraries were constructed, and sequencing was ultimately conducted on the Illumina MiSeq PE300 platform. The instestinal microbiome sequencing was completed by Shanghai Majorbio Technology Co., Ltd. The obtained raw data underwent quality control filtering to remove low‐quality sequences, and then, the clustering and classification operation was carried out to operational taxonomic units (OTUs). Subsequently, species annotation and classification were carried out based on the reference database (Greengenes: http://greengenes.secondgenome.com/ and SILVA: http://www.arb-silva.de), the diversity of microbial communities was analyzed (α‐diversity, such as Shannon, Simpson, ace, and chao index and β‐diversity, such as PCoA analysis), and the potential functions of microorganisms were annotated and interpreted through functional prediction tools (PICRUSt2: http://huttenhower.sph.harvard.edu/galaxy and KEGG: http://www.genome.jp/kegg/).

### 2.11. Statistic Analysis

All raw data are expressed as mean ± standard deviation (SD). Normality and homogeneity of variance were assessed for the original dataset. Then, one‐way ANOVA was used for significance analysis. Multiple comparisons were made on the average values of the treatment groups through the Tukey test, and the orthogonal polynomial comparison method was employed analyze the data trends to determine whether the experimental results conformed to the linear and/or quadratic polynomial trends. A statistically significant difference was defined as a *p*‐value less than 0.05.

## 3. Results

### 3.1. Effects of Dietary Rutin Supplementation on the Growth Performance of Tilapia Fed the Test Diets

The growth performance of tilapia after being fed with experimental diets for 10 weeks is presented in Table 2. Compared with C0, there was no significant difference in growth performance in R0 (*p* > 0.05). Compared with R0, FCR in R1 and R2 showed a downward trend, but no significant difference was observed (*p*>0.05), and there were no significant differences in the other growth indicators either (*p*>0.05).

**Table 2:** Growth performance of tilapia fed test diets for 10 weeks.

Group	C0	R0	R1	R2	*p*‐Value		
					ANOVA	Linear	Quadratic
IBW (g)	375.82 ± 13.59	369.92 ± 3.18	367.33 ± 5.44	372.92 ± 13.76	0.763	0.660	0.550
FBW (g)	757.41 ± 28.51	731.12 ± 18.94	771.55 ± 30.80	720.70 ± 13.62	0.105	0.385	0.552
WGR (%)^a^	101.90 ± 15.12	97.63 ± 3.74	110.14 ± 10.92	93.35 ± 3.48	0.254	0.650	0.567
FCR^b^	2.09 ± 0.17	2.08 ± 0.09	1.95 ± 0.14	2.01 ± 0.11	0.506	0.243	0.465
SR (%)^c^	100.00 ± 0.00	96.67 ± 5.77	96.67 ± 2.89	95.00 ± 5.00	0.532	0.150	0.352
SGR (%/day)^d^	1.00 ± 0.11	0.97 ± 0.03	1.06 ± 0.08	0.94 ± 0.03	0.240	0.638	0.557
CF (g /cm)^e^	4.05 ± 0.29	4.19 ± 0.16	4.25 ± 0.03	4.27 ± 0.06	0.445	0.113	0.243
HSI (%)^f^	1.98 ± 0.14	2.00 ± 0.09	2.00 ± 0.13	2.09 ± 0.24	0.823	0.375	0.630
VSI (%)^g^	9.36 ± 0.60	8.72 ± 0.69	8.88 ± 0.75	9.54 ± 0.24	0.352	0.687	0.179

*Note:* C0, R0, R1, and R2 represent the 0% fava bean supplementation group, the 60% fava bean supplementation group, the 60% fava bean supplementation group with 150 mg/kg rutin, and the 60% fava bean supplementation group with 300 mg/kg rutin. Data were presented as the means ± SD (*n* = 3).

^a^Weight gain rate (%) = (Final mean weight – initial mean weight)/initial mean weight × 100.

^b^Feed conversion ratio = Dry feed intake (g)/wet weight gain (g).

^c^Survival rate (SR) = 100 × (number of final fish/number of initial fish).

^d^Specific growth rate (%/day) = 100 × ln (final weight/initial weight)/days.

^e^Condition factor (g/cm^3^) = (Body weight, g)/(body length, cm)^3^× 100.

^f^Hepatosomatic index = 100 × (liver weight, g)/(body weight, g).

^g^Viscerosomatic index = 100 × (viscera weight, g)/(body weight, g).

### 3.2. Effects of Dietary Rutin on the Blood Components of Tilapia Fed the Test Diets

The blood cell composition of tilapia after being fed with experimental diets for 10 weeks is presented in Table 3. Compared with C0, the number of red blood cells (RBCs), hemoglobin (HGB), and hematocrit (HCT) in R1 were significantly decreased (*p* < 0.05), while other indicators showed no significant changes (*p* > 0.05). Compared with the R0, the levels of HGB, mean corpuscular volume (MCV), mean corpuscular HGB (MCH), MCH concentration (MCHC), and thrombocytocrit (PCT) in the R1 increased significantly (*p* < 0.05), while RBC volume distribution width (RDW) decreased significantly (*p* < 0.05). In R2, RBC and HGB increased significantly compared with R0 (*p* < 0.05) and no statistical differences were observed in the remaining indicators among all groups (*p* > 0.05).

**Table 3:** Whole blood cell composition analysis of tilapia fed test diets for 10 weeks.

Groups	C0	R0	R1	R2	*p*‐Value		
					ANOVA	Linear	Quadratic
WBC (10^9^/L)	215.67 ± 6.37^a^	224.27 ± 5.51^a^	219.93 ± 4.45^a^	222.97 ± 5.42^a^	0.297	0.261	0.394
RBC (10^12^/L)	1.51 ± 0.11^b^	1.26 ± 0.09^a^	1.20 ± 0.02^a^	1.52 ± 0.01^b^	0.001	0.934	<0.001
HGB (g /L)	126.00 ± 13.11^b^	88.67 ± 7.51^a^	116.33 ± 1.53^b^	130.67 ± 3.06^b^	0.001	0.402	0.013
HCT (%)	25.87 ± 0.91^b^	21.00 ± 1.37^a^	22.90 ± 2.14^ab^	24.80 ± 0.46^b^	0.011	0.836	0.014
MCV (fl)	167.57 ± 1.6^a^	165.57 ± 3.95^a^	177.00 ± 0.89^b^	169.87 ± 3.57^ab^	0.005	0.175	0.283
MCH (pg)	74.80 ± 4.09^a^	77.67 ± 2.34^ab^	91.30 ± 4.13^c^	86.33 ± 2.17^bc^	0.001	0.005	0.009
MCHC (g /L)	487.67 ± 17.01^a^	470.00 ± 4.58^a^	568.67 ± 8.08^b^	485.00 ± 7.55^a^	<0.001	0.421	0.291
RDW(%)	39.27 ± 1.48^ab^	41.37 ± 0.80^b^	38.87 ± 0.35^a^	40.83 ± 0.40^ab^	0.023	0.546	0.839
PLTs (10^9^/L)	72.33 ± 4.51^a^	63.67 ± 3.79^a^	66.0 ± 2.00^a^	68.67 ± 6.66^a^	0.192	0.540	0.125
MPV (fL)	6.27 ± 0.12^a^	6.43 ± 0.06^a^	6.20 ± 0.10^a^	6.40 ± 0.26^a^	0.286	0.716	0.928
PDW	18.73 ± 0.35^a^	18.73 ± 0.15^a^	18.63 ± 0.23^a^	18.77 ± 0.12^a^	0.902	1.000	0.875
PCT (%)	0.04 ± 0.00^a^	0.05 ± 0.01^a^	0.07 ± 0.01^b^	0.05 ± 0.00^a^	0.006	0.283	0.035

*Note:* C0, R0, R1, and R2 represent the 0% fava bean supplementation group, the 60% fava bean supplementation group, the 60% fava bean supplementation group with 150 mg/kg rutin, and the 60% fava bean supplementation group with 300 mg/kg rutin. Data were presented as the means ± SD (*n* = 3). Different superscript letters indicated statistical differences (*p* < 0.05). PCT, thrombocytocrit; RDW, red cell distribution width; RBC, red blood cell count; WBC, white blood cell count.

Abbreviations: HCT, hematocrit; HGB, hemoglobin concentration; MCH, mean corpuscular hemoglobin; MCHC, mean corpuscular hemoglobin concentration; MCV, mean corpuscular volume; MPV, mean platelet volume; PDW, platelet distribution width; PLTs, platelets.

### 3.3. Effects of Dietary Rutin on the Serum Biochemical Indicators of Tilapia Fed the Test Diets

As indicated in Table 4, the serum activities of AST and ALT were highest in R0, whereas the levels of TP, ALB, TG, T‐CHO, and ALP were significantly lower than those in Group C0 (*p* < 0.05). Compared with R0, R1, and R2 exhibited reduced activities of AST and ALT, as well as lower levels of TP and ALB ( *p* < 0.05). Among the three groups, the levels of TG, GLU, and ALP in R2 were the highest, with all values significantly different (*p* < 0.05).

**Table 4:** Serum biochemical levels of tilapia fed test diets for 10 weeks.

Groups	C0	R0	R1	R2	*p*‐Value		
					ANOVA	Linear	Quadratic
TP (g/ L)	36.58 ± 0.34^c^	29.14 ± 0.89^b^	27.47 ± 0.46^a^	28.04 ± 0.23^ab^	<0.001	0.001	<0.001
ALB (g/ L)	17.8 ± 0.36^c^	15.43 ± 0.35^b^	13.9 ± 0.56^a^	14.40 ± 0.56^ab^	<0.001	0.001	<0.001
TG (nmol/L)	3.74 ± 0.26^b^	1.96 ± 0.50^a^	3.11 ± 0.15^b^	1.29 ± 0.23^a^	<0.001	0.011	0.047
GLU (nmol /L)	3.99 ± 0.60^a^	4.61 ± 0.42^ab^	5.3 ± 0.15^b^	4.04 ± 0.34^a^	0.015	0.648	0.020
T‐BIL‐V (µmol/L)	6.42 ± 0.37^b^	5.39 ± 0.56^ab^	4.89 ± 0.27^a^	6.24 ± 0.39^b^	0.006	0.612	0.003
ALP (U/L)	26.13 ± 1.82^b^	19.30 ± 3.12^a^	22.63 ± 1.4^ab^	18.73 ± 1.15^a^	0.007	0.031	0.077
AST (U/L)	29.00 ± 4.59^a^	57.33 ± 1.86^b^	57.20 ± 10.38^b^	31.20 ± 7.02^a^	0.001	0.879	<0.001
ALT (U/L)	22.40 ± 1.73^a^	32.23 ± 0.45^b^	21.27 ± 1.52^a^	23.10 ± 0.82^a^	<0.001	0.490	0.278
T‐CHO (nmol /L)	3.61 ± 0.11^b^	2.52 ± 0.23^a^	2.89 ± 0.28^a^	2.79 ± 0.18^a^	0.001	0.072	0.015

*Note:* C0, R0, R1, and R2 represent the 0% fava bean supplementation group, the 60% fava bean supplementation group, the 60% fava bean supplementation group with 150 mg/kg rutin, and the 60% fava bean supplementation group with 300 mg/kg rutin. Data were presented as the means ± SD (*n* = 3). Different superscript letter indicated statistical differences (*p* < 0.05). T‐BIL‐V, total bilirubin.

Abbreviations: ALB, albumin; ALP, alkaline phosphatase; ALT, alanine aminotransferase; AST, aspartate aminotransferase; GLU, glucose; T‐CHO, total cholesterol; TG, triglyceride; TP, total protein.

### 3.4. Effects of Dietary Rutin on Muscle of Tilapia Fed the Test Diets

#### 3.4.1. Nutrient Compositions of Muscle

As shown in Table 5, compared with C0, the crude lipid level in the muscle of R0 was significantly increased (*p* < 0.05), while there were no obvious changes in moisture, crude protein and ash (*p* > 0.05). Compared with the R0, the crude protein in the R1 and R2 increased significantly (*p* < 0.05), while there were no significant changes in moisture, crude fat and ash (*p* > 0.05). No differences in various indicators between R1 and R2 were observed (*p* > 0.05).

**Table 5:** Muscle proximate composition of tilapia fed test diets for 10 weeks (%).

Groups	C0	R0	R1	R2	*p*‐Value		
					ANOVA	Linear	Quadratic
Moisture	76.47 ± 1.17	76.33 ± 0.26	75.72 ± 0.87	75.57 ± 0.57	0.463	0.104	0.287
Crude protein	19.48 ± 0.18^a^	19.49 ± 0.21^a^	21.11 ± 0.46^b^	21.02 ± 0.69^b^	0.002	0.002	0.002
Crude lipid	1.67 ± 0.03^a^	1.8 ± 0.04^b^	1.78 ± 0.02^b^	1.76 ± 0.03^b^	0.004	0.074	0.003
Ash	1.18 ± 0.03	1.19 ± 0.02	1.23 ± 0.01	1.21 ± 0.02	0.067	0.043	0.085

*Note:* C0, R0, R1, and R2 represent the 0% fava bean supplementation group, the 60% fava bean supplementation group, the 60% fava bean supplementation group with 150 mg/kg rutin, and the 60% fava bean supplementation group with 300 mg/kg rutin. Data were presented as the means ± SD (*n* = 3). Different superscript letters indicated statistical differences (*p* < 0.05).

#### 3.4.2. Muscle Texture Characteristics

The muscle texture characteristics of tilapia are shown in Table 6. R0 observed significant improvements in hardness, springiness, cohesiveness, gumminess, chewiness, and resilience of tilapia muscles, as well as significant decreases in cohesiveness and stringiness (*p* < 0.05). The hardness, gumminess, and resilience of the R1 and R2 increased significantly compared with the R0 (*p* < 0.05). Springines, cohesiveness, and chewiness significantly improved with the increase of rutin concentration (*p* < 0.05). Adhesiveness significantly decreased in the R1, but significantly increased in the R2 group (*p* < 0.05).

**Table 6:** Muscle texture mechanical properties of tilapia fed test diets for 10 weeks.

Groups	C0	R0	R1	R2	*p*‐Value		
					ANOVA	Linear	Quadratic
Hardness (g)	1195.33 ± 90.98^a^	1783.83 ± 81.31^b^	2173.33 ± 134.38^c^	2243.33 ± 131.93^c^	<0.001	<0.001	<0.001
Adhesiveness, (g sec)	7.43 ± 0.25^d^	3.74 ± 0.25^b^	2.87 ± 0.39^a^	4.22 ± 0.19^c^	<0.001	<0.001	<0.001
Springiness (mm)	0.34 ± 0.02^a^	0.39 ± 0.01^b^	0.43 ± 0.02^c^	0.53 ± 0.02^d^	<0.001	<0.001	<0.001
Cohesiveness	0.26 ± 0.01^a^	0.31 ± 0.01^b^	0.40 ± 0.03^c^	0.46 ± 0.02^d^	<0.001	<0.001	<0.001
Gumminess (g)	311.68 ± 48.81^a^	558.93 ± 32.45^b^	897.59 ± 58.48^c^	974.29 ± 58.17^c^	<0.001	<0.001	<0.001
Chewiness (g)	105.75 ± 23.56^a^	213.23 ± 20.49^b^	391.65 ± 51.54^c^	523.72 ± 44.17^d^	<0.001	<0.001	<0.001
Resilience	0.12 ± 0.00^a^	0.19 ± 0.01^b^	0.26 ± 0.01^c^	0.27 ± 0.01^c^	<0.001	<0.001	<0.001
Stringiness	1.27 ± 0.13^b^	1.05 ± 0.06^a^	0.99 ± 0.08^a^	1.10 ± 0.09^a^	<0.001	0.023	<0.001

*Note:* C0, R0, R1, and R2 represent the 0% fava bean supplementation group, the 60% fava bean supplementation group, the 60% fava bean supplementation group with 150 mg/kg rutin, and the 60% fava bean supplementation group with 300 mg/kg rutin. Data were presented as the means ± SD (*n* = 3). Different superscript letters indicated statistical differences (*p* < 0.05).

#### 3.4.3. Histomorphological Changes of Muscle

As shown in Figure 1A, B, compared with Group C0, the cross‐sectional area and diameter of muscle fibers in R0 were significantly reduced (*p* < 0.05), whereas the density of muscle fibers was significantly increased (*p* < 0.05). Compared with R0, the cross‐sectional area and diameter of muscle fibers in R1 and R2 exhibited a significant increase (*p* < 0.05), while the muscle fiber density experienced a significant decrease (*p* < 0.05), and there was no statistical difference between those two groups (*p* > 0.05).

Figure 1Physiological changes in tilapia muscle after 10 weeks of feeding with the test diets. (A) H.E. staining. (B) Quantification of muscle fibers. (C) Muscle antioxidant indicators. (D) Muscle hydroxyproline levels. MF, muscle fiber; MFLD, muscle fiber long diameter; MFSD, muscle fiber short diameter. C0, R0, R1 and R2 represent the 0% fava bean supplementation group, the 60% fava bean supplementation group, the 60% fava bean supplementation group with 150 mg/kg rutin, and the 60% fava bean supplementation group with 300 mg/kg rutin. Data were presented as the means ± SD (*n* = 3). Different superscript letters indicated statistical differences (*p* < 0.05).

**Figure:**
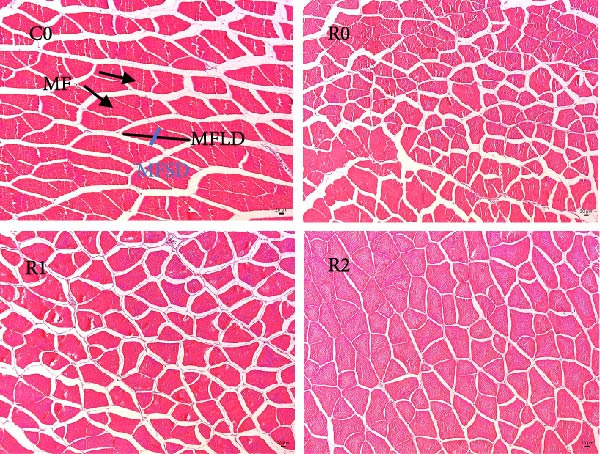
(A)

**Figure:**
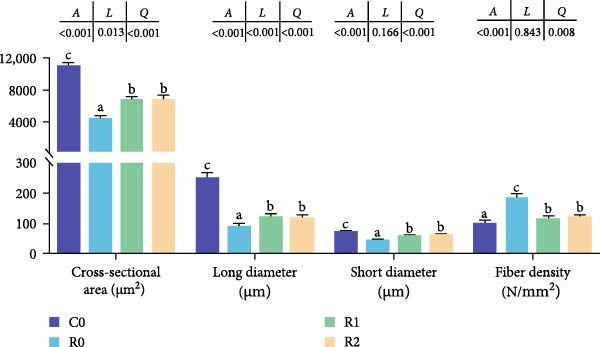
(B)

**Figure:**
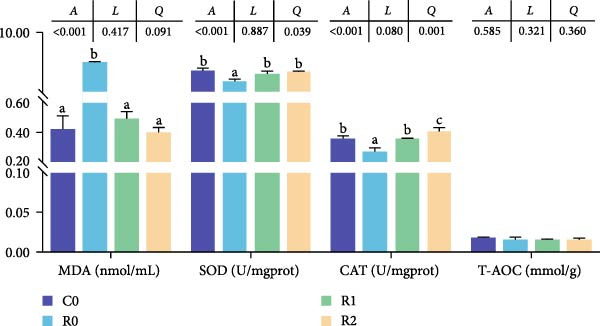
(C)

**Figure:**
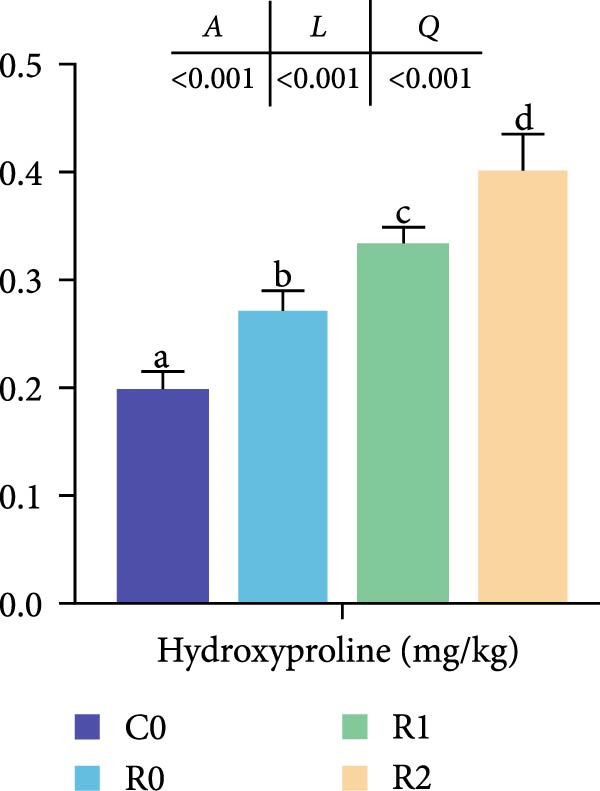
(D)

#### 3.4.4. AOC of Muscles

As shown in Figure 1C, after feeding 60% fava beans diet (C0), tilapia muscle exhibited significantly depressed SOD and CAT activities (*p* < 0.05) alongside a marked elevation in MDA content (*p* < 0.05). After adding 150–300 mg/kg rutin to the 60% fava beans diet (R1 and R2), muscle SOD and CAT activities demonstrated restoration relative to the R0 (*p* < 0.05), accompanied by a marked decrease in MDA concentration (*p* < 0.05). No significant variations in T‐AOC were detected across the four experimental groups (*p* > 0.05).

#### 3.4.5. Hydroxyproline Levels of Muscles

As shown in Figure 1D, the hydroxyproline level in R0 was significantly higher than that in C0 (*p* < 0.05). The hydroxyproline levels in R1 and R2 were significantly increased compared to R0 and were proportional to the added amount of rutin (*p* < 0.05).

### 3.5. Effects of Dietary Rutin on Liver Health of Tilapia Fed the Test Diets

#### 3.5.1. Histomorphological Changes and Lipid Deposition of Liver

Compared to the C0, the R0 showed severe vacuolization of liver cells and displacement of cell nuclei (Figure 2A). The results of Masson staining and Oil Red O staining showed significant liver fibrosis and degeneration in the R0 (Figure 2B, D; *p* < 0.05), along with the formation of numerous lipid droplets (Figure 2C, E; *p* < 0.05). In the R1 and R2, liver cells were arranged relatively neatly, with reduced vacuolization and nuclear displacement compared to the R0, and the degree of liver fibrosis and the proportion of lipid droplets were significantly decreased (*p* < 0.05). The detection of TG and T‐CHO levels in liver revealed that compared with Group C0, the contents of TG and T‐CHO in R0 increased significantly (*p* < 0.05), while the contents of TG in R1 and R2 decreased significantly compared with R0 (*p* < 0.05), and there was no significant difference in the contents of T‐CHO (Figure 2F; *p* > 0.05).

Figure 2Physiological changes in tilapia liver after 10 weeks of feeding with the test diets. (A) H.E. staining. (B) Masson staining. (C) Oil red O staining. (D) Proportion of fibrotic area (blue part). (E) Proportion of lipid droplet area (red part). (F) Total triglyceride (TG) and total cholesterol (TC) content in the liver. (G) Liver antioxidant indicators. CN, cell nucleus; CV, cellular vacuoles; HC, hepatocyte cell. C0, R0, R1, and R2 represent the 0% fava bean supplementation group, the 60% fava bean supplementation group, the 60% fava bean supplementation group with 150 mg/kg rutin, and the 60% fava bean supplementation group with 300 mg/kg rutin. Data were presented as the means ± SD (*n* = 3). Different superscript letters indicated statistical differences (*p* < 0.05).

**Figure:**
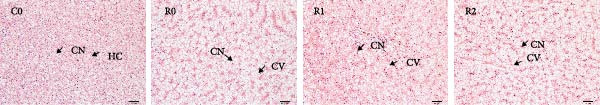
(A)

**Figure:**
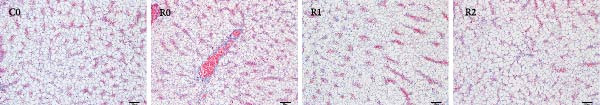
(B)

**Figure:**
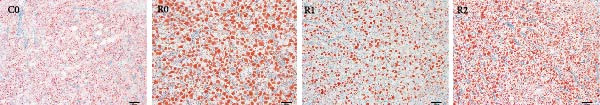
(C)

**Figure:**
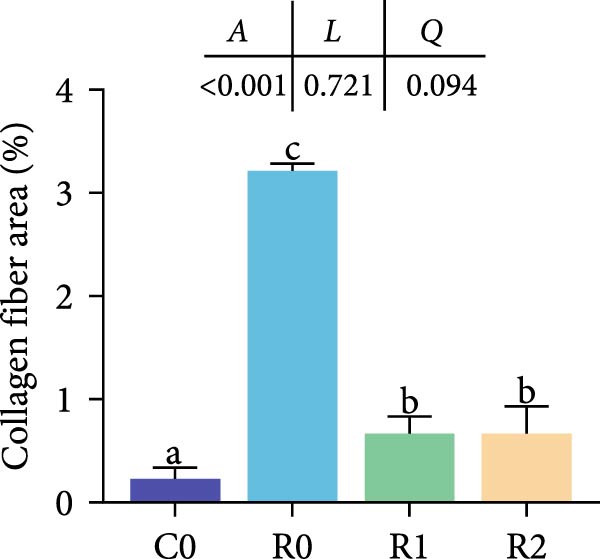
(D)

**Figure:**
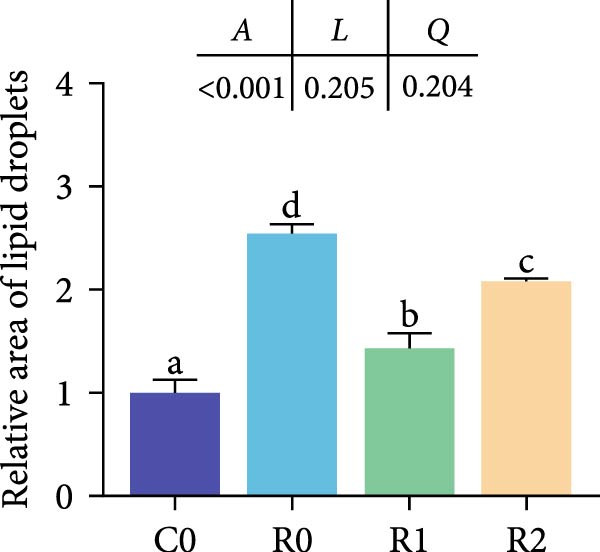
(E)

**Figure:**
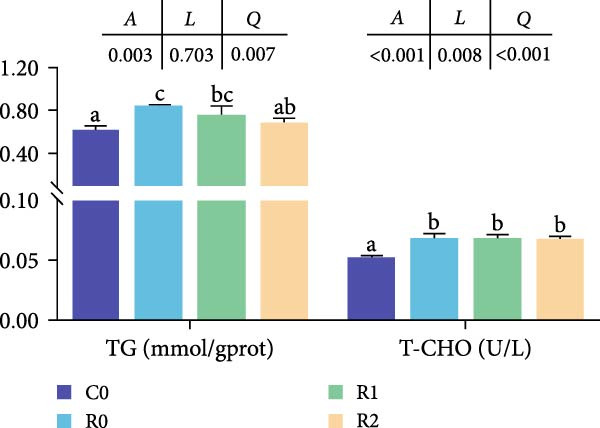
(F)

**Figure:**
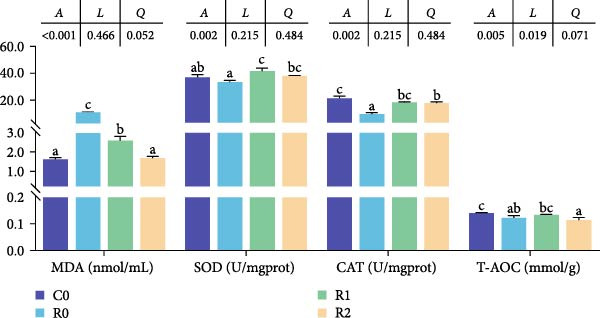
(G)

#### 3.5.2. AOC of Liver

As shown in Figure 2G, the level of CAT and T‐AOC in R0 decreased significantly compared with C0 (*p* < 0.05), while MDA level increased significantly (*p* < 0.05). Compared with R0, CAT and SOD levels in R1 and R2 were increased (*p* < 0.05), while MDA level was inversely proportional to rutin concentration (*p* < 0.05). The level of T‐AOC showed no significant change among R0, R1, and R2 (*p* > 0.05).

### 3.6. Effects of Dietary Rutin on Intestinal Health of Tilapia Fed the Test Diets

#### 3.6.1. Histomorphological Changes of Intestine

As depicted in Figure 3A, B, compared to C0, the intestinal villi in R0 were shortened (*p* < 0.05), exhibiting a notable reduction in muscularis thickness and a decrease in villus number (*p* < 0.05). After administering 150–300 mg/kg rutin, the musclaris thickness and villus number in R1 and R2 increased significantly (*p* < 0.05), while no distinction existed among the two groups (*p* > 0.05), the length of intestinal villi increased with the increase of rutin supplementation (*p* < 0.05). Compared to Group R0, the width of villi did not show significant changes in R1, but it increased significantly in R2 (*p* < 0.05).

Figure 3Physiological changes in tilapia intestine after 10 weeks of feeding with the test diets. (A) H.E. staining. (B) Quantification of intestinal villi. The levels of SOD (C), MDA (D), LPS (E), and trypsin (F) in the intestine. IV, intestinal villi; MT, musclaris thickness; VL, villus length; VW, villus width. C0, R0, R1, and R2 represent the 0% fava bean supplementation group, the 60% fava bean supplementation group, the 60% fava bean supplementation group with 150 mg/kg rutin, and the 60% fava bean supplementation group with 300 mg/kg rutin. Data were presented as the means ± SD (*n* = 3). Different superscript letters indicated statistical differences (*p* < 0.05).

**Figure:**
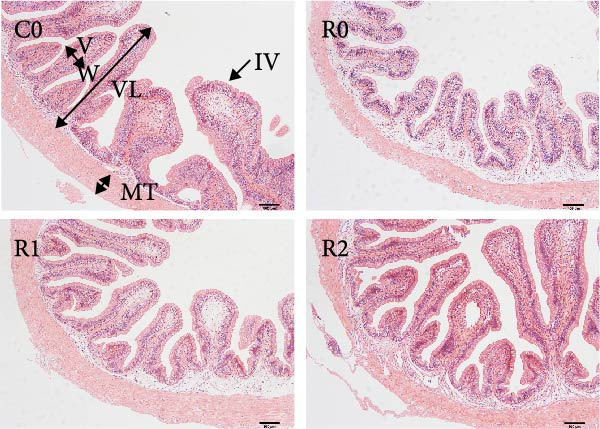
(A)

**Figure:**
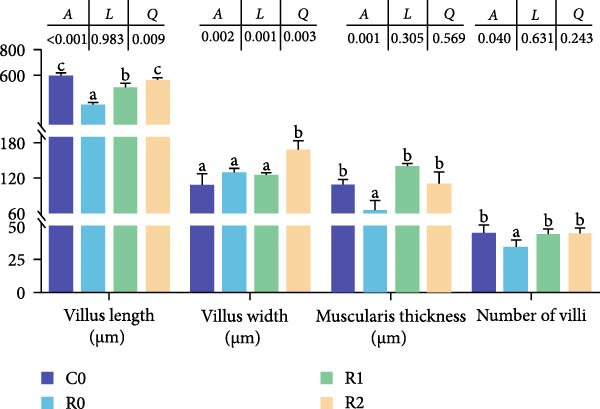
(B)

**Figure:**
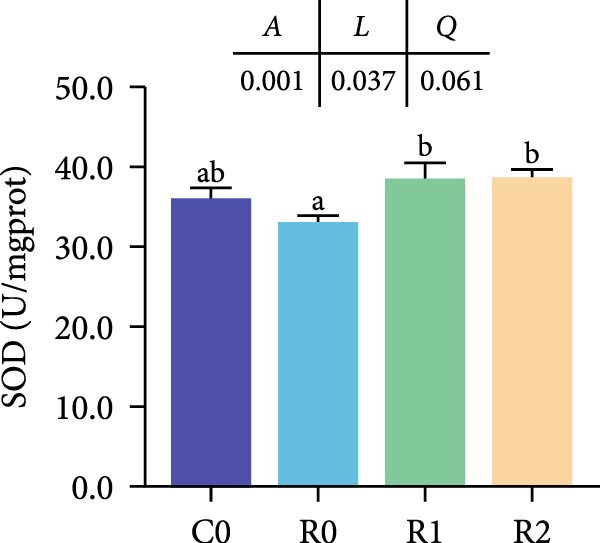
(C)

**Figure:**
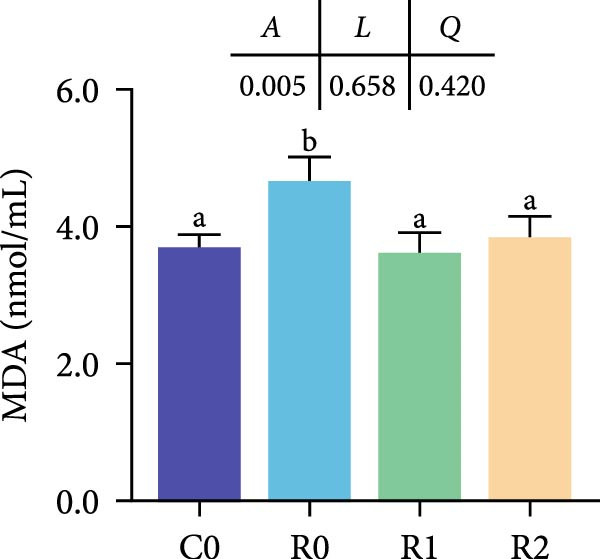
(D)

**Figure:**
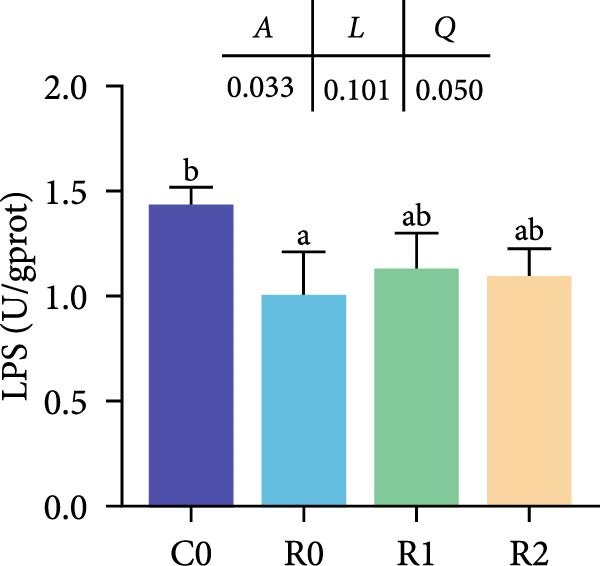
(E)

**Figure:**
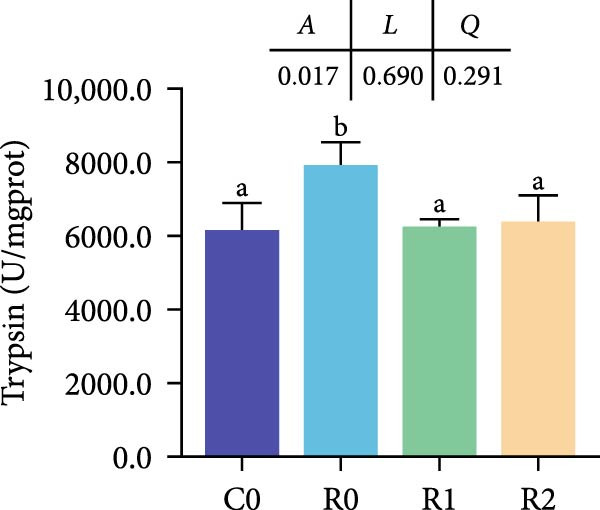
(F)

#### 3.6.2. Activity of Antioxidant Enzymes and Digestive Enzymes in the Intestine

Figure 3C–F show the activity or content of MDA, SOD, LPS, and trypsin in the intestine. The results indicated that MDA levels and trypsin activity significantly increased in the R0 (*p* < 0.05), while the LPS activity was significantly inhibited (*p* < 0.05). Compared to R0, the MDA content and trypsin activity were significantly reduced in the R1 and R2 (*p* < 0.05), while SOD activity increased significantly (*p* < 0.05). There was an upward trend in LPS activity, but it lacked statistical significance (*p* > 0.05).

#### 3.6.3. Intestinal Microbiota Composition

A total of 1,273,005 sequences were obtained from the analysis of the intestinal microbiome, with an average length of 454 bp. Analysis of OTU showed that there were 2560 common OTU in the four groups of intestinal microorganisms, and the specific OTU in C0, R0, R1, and R2 groups were 1015, 1106, 2115, and 910, respectively (Figure 4A). The results of the ɑ‐diversity analysis indicated that, in comparison with C0, the Simpson index for R0 was significantly reduced (*p* < 0.05), whereas the other indexes did not exhibit significant differences (*p* > 0.05). Compared with R0, the Simpson index decreased in R1 (*p* < 0.05), while the Ace index and Chao index increased significantly (*p* < 0.05); all indexes in R2 showed no significant changes (Figure 4B–E). The differences in the intestinal microbiota of tilapia at the phylum level were shown in Figure 4F. Among them, the top four with the highest relative abundance were *Proteobacteria*, *Actinobacteriota*, *Bacteroidota*, and *Firmicutes*. At the genus level (Figure 4G), the top four with the highest relative abundance were *Muribaculaceae_unclassified*, *Rhodobacteraceae*, *Chloroplast_unclassified*, and *Rothia*. Figure 4H shows the enrichment of the top 20 intestinal microbial communities at genus level in all groups, and their interaction is shown in Figure 4I.

Figure 4Intestinal microbiome analysis of tilapia after 10 weeks of feeding with the test diets. (A) Venn analysis of operational taxonomic units (OTUs) in each group. (B–E) Alpha diversity analysis. (F) Composition of microbiota at the phylum level in each group. (G) Composition of microbiota at the genus level in each group. (H) Heatmap analysis of the top 20 abundant genera. (I) Interactions among genera at the genus level. (J) Abundance proportion of the top four abundant genera in each group. C0, R0, R1, and R2 represent the 0% fava bean supplementation group, the 60% fava bean supplementation group, the 60% fava bean supplementation group with 150 mg/kg rutin, and the 60% fava bean supplementation group with 300 mg/kg rutin. Different superscript letters indicated statistical differences (*p* < 0.05).

**Figure:**
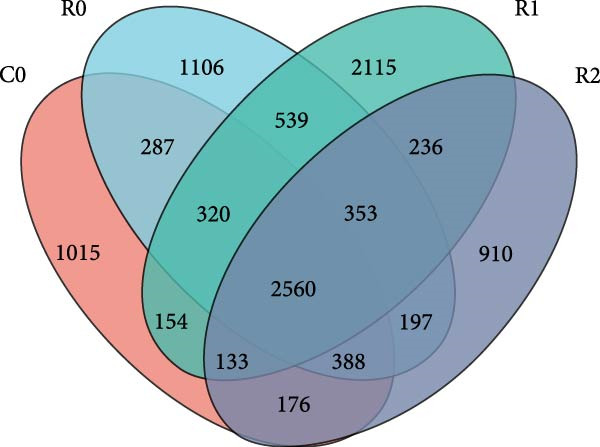
(A)

**Figure:**
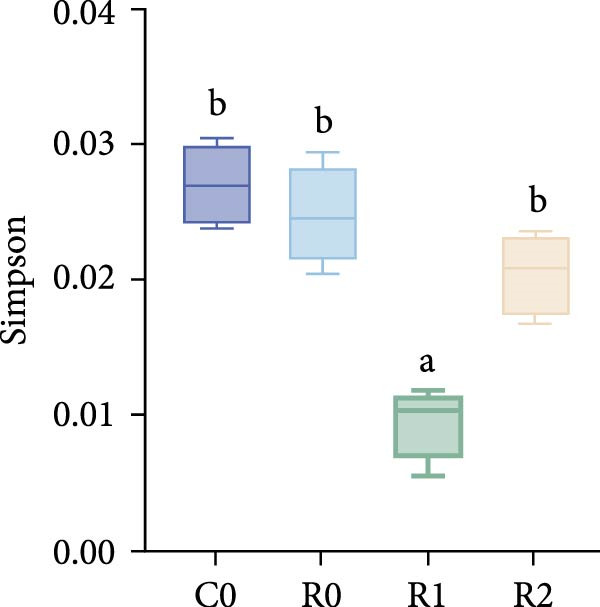
(B)

**Figure:**
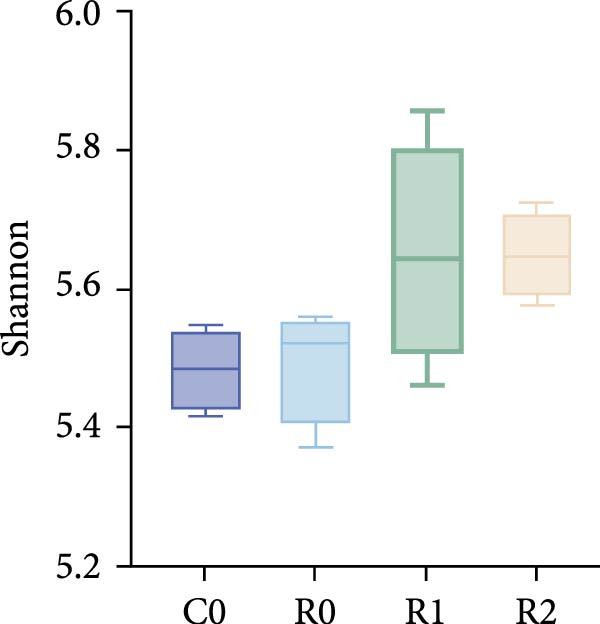
(C)

**Figure:**
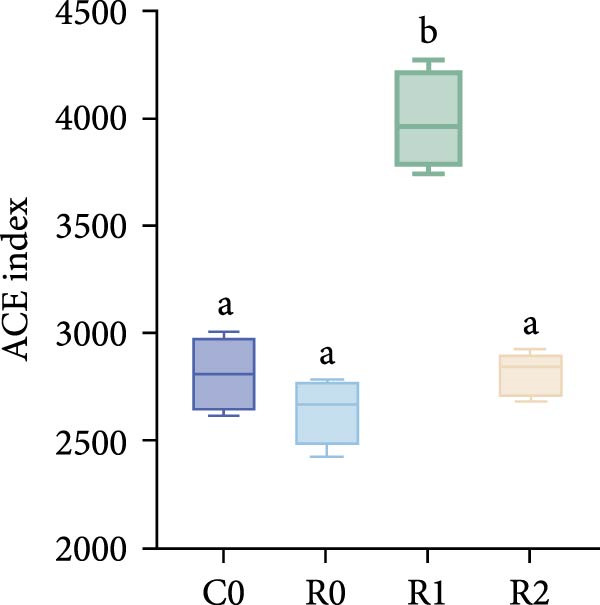
(D)

**Figure:**
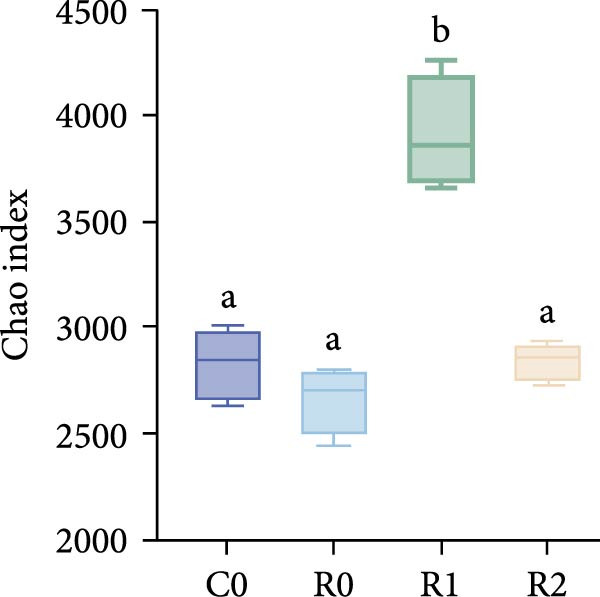
(E)

**Figure:**
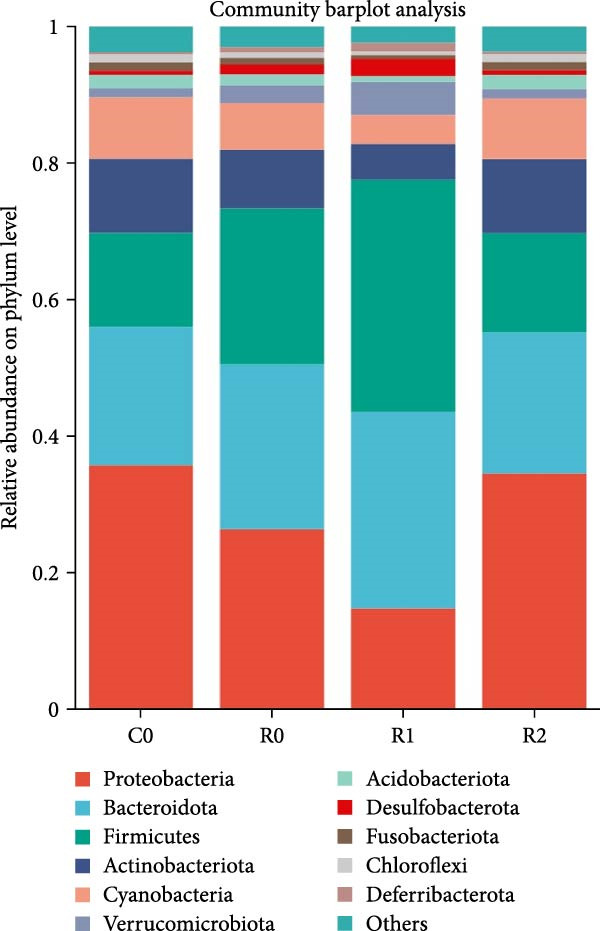
(F)

**Figure:**
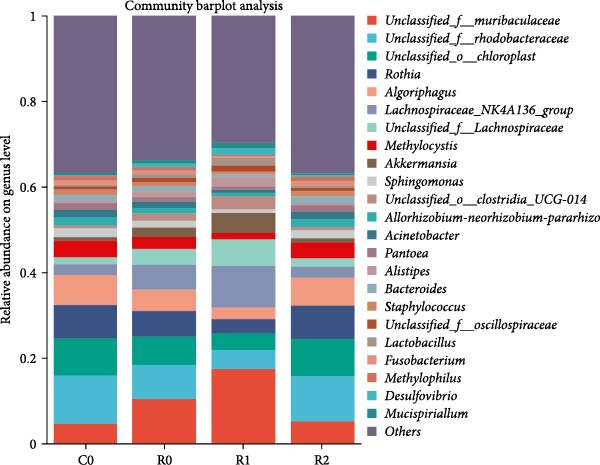
(G)

**Figure:**
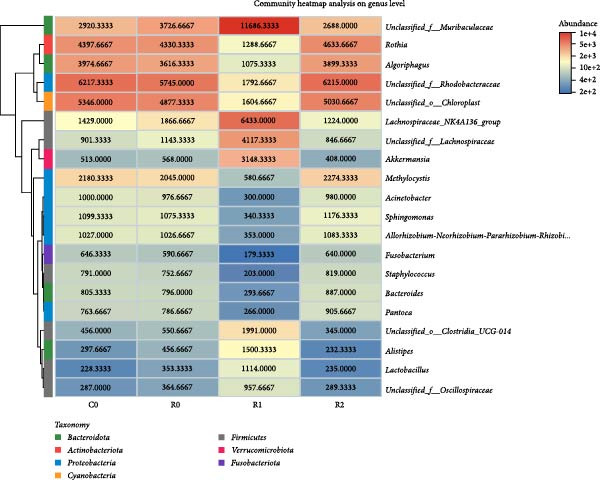
(H)

**Figure:**
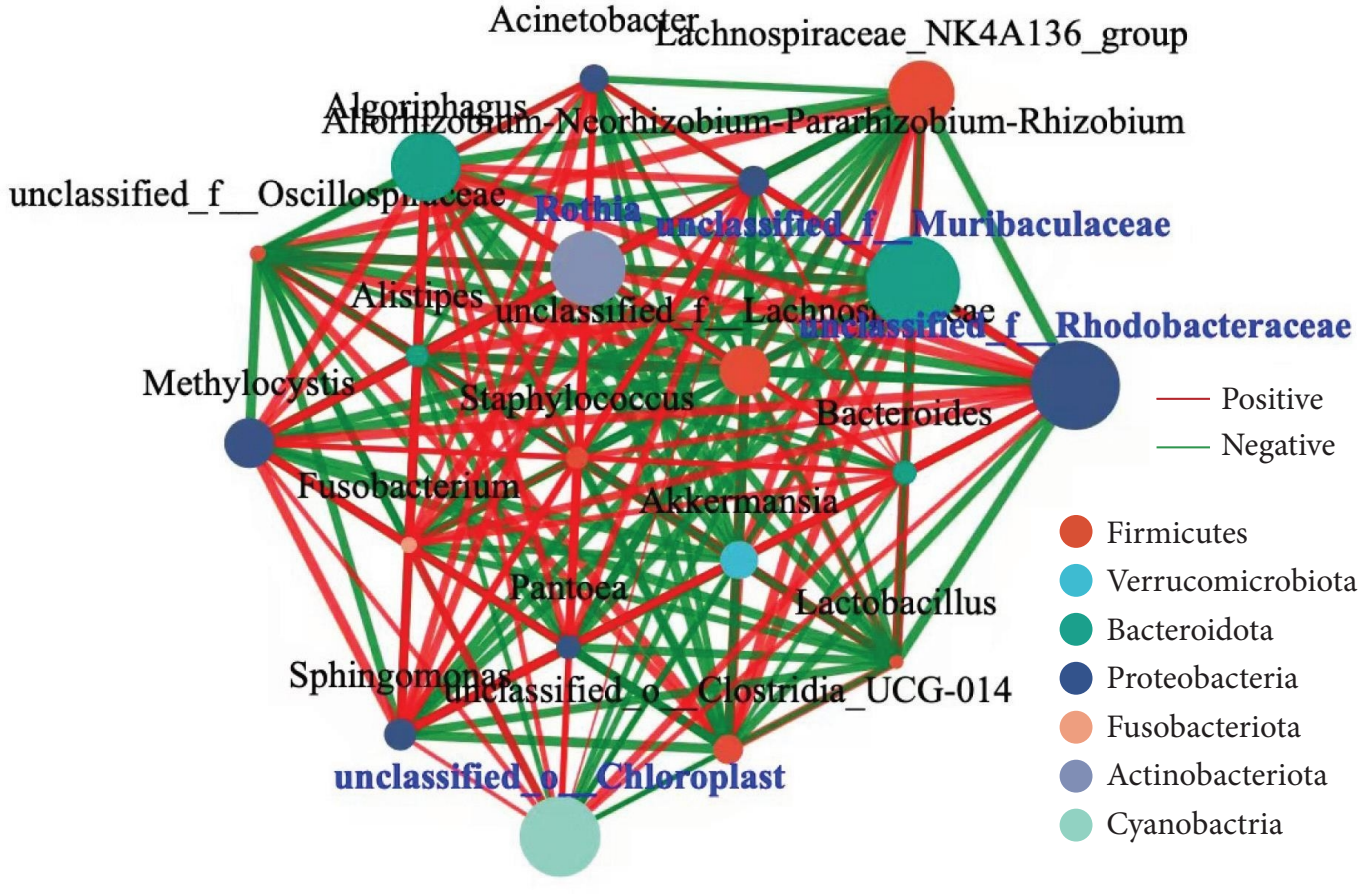
(I)

**Figure:**
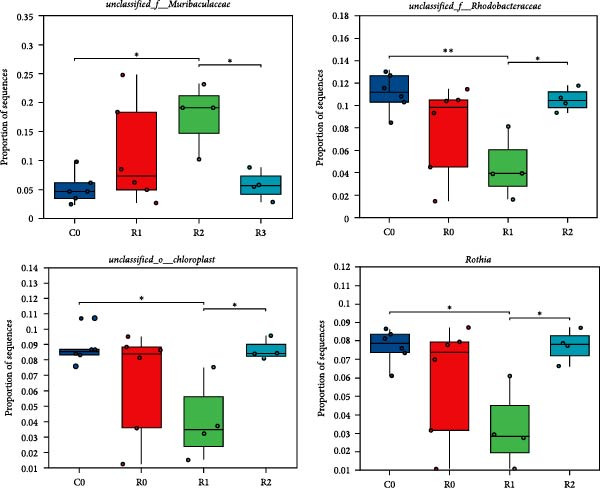
(J)

## 4. Discussion

Rutin has been proven to have obvious antioxidant and anti‐inflammatory capabilities, but it is still unknown whether it can alleviate hepatointestinal damage in tilapia induced by fava beans. This study assessed the impact of supplementing 60% fava bean feed with 150 and 300 mg/kg of rutin on the growth performance, muscle texture characteristics, and liver and intestinal health of tilapia. In terms of growth performance, the additional rutin had no negative effect on the survival rate and growth performance of tilapia.

The composition of blood cells can be used to evaluate the health status and metabolic strength of fish, offering reference value for understanding various physiological processes of fish and potential effects on blood‐related aspects [14]. The changes in blood parameters in this study reflected that the addition of fava beans and rutin had a significant impact on blood cell composition and hematopoietic tissue. The main functions of fish RBCs are to transport O_2_ and CO_2_, as well as to buffer the acidic and alkaline substances produced during the physiological processes of the body and maintain ionic balance [15, 16]. Clinically, the three indicators of HGB, RBC, and HCT are interrelated. A high level of these three indicators may be caused by inflammation or hypoxia, while a low level may be due to malnutrition and anemia [17, 18]. The three indicators of MCV, MCH, and MCHC are important reference indicators for the differential diagnosis of anemia. An increase in these values may indicate malnutrition anemia or hemorrhagic anemia, whereas a decrease could suggest secondary anemia or hemolytic anemia [19]. This study found that the levels of HGB, RBC, and HCT in the blood of tilapia in Group R0 were significantly decreased, indicating that 60% fava bean feed would cause malnutrition in tilapia and lead to anemia symptoms. After adding 150 –300 mg/kg of rutin, the indicators of HGB, RBC, HCT, MCV, MCH, and MCHC in the blood of tilapia increased, suggesting that the addition of rutin can repair the nutritional anemia caused by fava beans and improve the health status of tilapia.

The core advantage of the crispy aquaculture method lies in its ability to effectively optimize the nutritional composition and texture characteristics of fish muscle. The proportion relationship of the main nutritional components of muscle fibers (crude protein, crude fat, moisture, and ash) directly determines their edible value. It is worth noting that protein, as a key structural component for the growth and development of animals, plays an irreplaceable biological role in physiological metabolism. The current data showed that rutin significantly increased the crude protein content of muscle in crispy tilapia and presented a negative regulatory trend of crude fat content. This phenomenon may be related to the dual metabolic regulatory mechanism of flavonoids: First, flavonoids promote the decomposition of fatty acids, reduce the proportion of proteins as energy supply substances, and thereby increasing the deposition of muscle proteins [20, 21]. Second, flavonoids can enhance the expression efficiency of intestinal amino acid transport carriers, thereby increasing the nitrogen deposition rate and the rate of muscle protein synthesis [22]. This conclusion was also supported by the serum biochemical results of this experiment. The concentration of TG and T‐CHO in the circulatory system increased dose‐dependently, while the levels of TP and ALB decreased significantly. Therefore, it is speculated that rutin can effectively reconstruct the energy metabolism pathway, prioritizing the energy generated by catabolism towards protein biosynthesis, and ultimately achieving the structural optimization of muscle nutritional quality. This might explain why the dietary rutin can enhance the body’s digestion and absorption of nutrients, promote amino acid transport, and increase the anabolism of muscle proteins.

Muscle texture is a sensory characteristic, which can be evaluated by hardness, chewing, elasticity, and other indicators [23]. The changes in dorsal muscle hardness and masticability are important indicators for evaluating muscle crisp, and muscle fibers are the main components of muscles and can directly reflect meat quality [24]. This study suggested that rutin further enhanced the hardness, gumminess, resilience, springiness, cohesiveness, and chewiness of the muscle, and the muscle fibers were arranged regularly and neatly with complete morphology, which suggested that 150 –300 mg/kg diarary rutin can further enhance the muscular texture characteristics of crispy tilapia. However, it is worth noting that, generally speaking, the smaller the cross‐sectional area of muscle fibers, the greater the density and the higher the muscle hardness [25], but different results have been obtained in the current study. In fact, apart from the morphology of muscle fibers, the content of collagen in muscles is also positively correlated with hardness [25]. Hydroxyproline is the signature amino acid of collagen. According to AOAC [12], the content of collagen is equal to the content of hydroxyproline multiplied by 8. The determination results of hydroxyproline in the muscle confirmed that the collagen content in R1 and R2 increased significantly, which might partially explain that R1 and R2 exhibited higher hardness compared to Group R0 when the cross‐sectional area of muscle fibers increased. Moreover, muscle AOC is also a significant factor influencing muscle quality. The findings of this study indicate that rutin mitigated the decrease in muscle AOC induced by fava beans, preserved normal cell metabolism, and diminished oxidative damage to muscle cells. Similar conclusions have also been drawn in the research on yellow catfish and rainbow trout [9, 10].

Liver is the primary organ of metabolism and can reflect the health state of the body. Findings from our laboratory indicated that long‐term consumption of fava beans can have adverse effects on the liver health of fish, possibly leading to liver damage and steatosis. In the current study, rutin alleviated liver damage induced by 60% dietary fava beans, reducing the degree of fibrotic degeneration and showed a significant dose‐dependent effect. TG and T‐CHO are the main components of lipids and are mainly synthesized and stored by the liver in fish. In human medicine, elevated levels of TG and T‐CHO may trigger nonalcoholic fatty liver disease (NAFLD) [26]. Rutin could improve lipid accumulation in the liver and alleviate lipid metabolism dysfunction in diabetic NAFLD through the AMPK/SREBP1 pathway [27]. In this study, the fat accumulation in the liver of tilapia was analyzed by staining liver slices with oil red O and detecting the contents of TG and T‐CHO. The results confirmed that rutin could inhibit liver fat accumulation induced by 60% dietary fava beans, which was consistent with the previous research results.

Serum biochemical indicators can also indirectly reflect changes in liver function status [28]. Liver function assessment in fish species frequently utilizes aspartate AST and ALT as principal enzymatic markers [29]. AST and ALT are mainly synthesized by the liver. When liver cells are damaged, or the permeability of the cell membrane increases, or the cells rupture, AST and ALT are released into the serum, leading to an elevation in serum transaminase levels. ALP is a nonspecific immune enzyme that plays a positive role in promoting the immunity of the body [30, 31]. In the current study, rutin reduced the increase in serum AST and ALT levels and the decrease in ALP levels caused by 60% dietary fava beans. The above results indicated that rutin can repair liver damage caused by fava beans via enhancing the AOC and regulating lipid metabolism levels of liver.

The intestine plays a vital role in digesting and absorbing nutrients. A healthy intestine can act as an immune barrier to resist the entry of external microorganisms, bacteria and toxic substances [32]. Intestinal villi play a crucial role in digesting and absorbing nutrients inside the intestinal tract. The rhythmic contraction and relaxation of the intestinal muscular layer cause intestinal peristalsis, pushing the chyme to move towards the distal end of the digestive tract [33]. Therefore, intestinal morphology can be used to assess the health and functional status of fish intestines [34]. In this study, after the addition of rutin, intestinal morphology was restored, with a significant increase in the length of intestinal villi and a noticeable thickening of the muscularis. This might indicated that the contact area between intestinal epithelial cells and nutrients increased and the digestive capacity was enhanced. Analysis of intestinal digestive enzymes revealed that the activity of intestinal LPS returned to its pre‐fava bean addition level after the addition of rutin. Meanwhile, rutin addition exhibited a marked elevation in SOD, reduced the accumulation of MDA, and enhanced the AOC of the intestine. This might be due to the unique pharmacological activity of rutin, which enhances the antioxidant defense mechanism of tilapia, reduces the damage to the intestinal tract caused by oxidative stress, thereby improving the intestinal morphology and enhancing the digestive capacity of the fish [35].

As an important microecosystem in the host, the intestinal microbiota of fish plays a key role in maintaining physiological homeostasis by constructing biological barriers [36]. The intestinal microbiota can produce bioactive substances through nutritional metabolism transformation, interact with intestinal epithelial cells to regulate mucosal immune function, and inhibit pathogen colonization through niche competition [37–39]. The homeostasis of the intestinal microbiota is closely related to α‐diversity. Generally, higher intestinal microbiota α‐diversity is associated with enhanced functional comprehensiveness, promoting ecosystem stability through improved physiological coordination [40]. In this study, 150 mg/kg rutin significantly increased the richness of the intestinal flora in tilapia. Existing studies have shown that the intestinal microbiota of healthy fish is predominantly composed of *Bacteroidetes* and *Fusobacteria*, whereas *Proteobacteria*, *Actinobacteriota*, and *Tenericutes* dominate in unhealthy specimens. *Bacteroidetes* and *Fusobacteria* are the dominant bacterial phyla in the intestines of healthy fish, while *Proteobacteria*, *Actinobacteriota*, and *Tenericutes* are the dominant bacterial phyla in the intestines of unhealthy fish [41]. Moreover, many bacteria belonging to the *Proteobacteria* and *Actinomycetes* phyla are pathogenic, linked to diseases such as enteritis and immune system disorders. The increase in their abundance may indicate an imbalance in the intestinal microbiota [42]. This study investigated the intestinal microbiota of tilapia and revealed significant phylum‐level alterations with 150 mg/kg rutin supplementation. The treatment notably diminished *Proteobacteria* and *Actinobacteriota*, while enhancing *Bacteroidetes* and *Firmicutes* predominance in intestinal communities. At the genus level, *Muribaculaceae* exhibited a significant rise in relative abundance, while the relative abundance of the *Rhodobacteraceae*, *Chloroplast*, and *Rothia* decreased significantly. As a member of the Bacteroidetes phylum, Muribaculaceae can compete with pathogenic bacteria for resources by occupying ecological niches on the intestinal tract, thereby inhibiting the growth of pathogenic bacteria. Meanwhile, *Muribaculaceae* can degrade complex polysaccharides such as starch in the intestinal tract into monosaccharides and short‐chain fatty acids [43]. Smith et al. [44] and Ormerod et al. [45] have shown that one of the short‐chain fatty acids produced by the *Muribaculaceae* family is propionic acid. Research on turbot has revealed that propionic acid can promote the growth performance and increase the expression of intestinal tight junction proteins [46]. Studies on Nile tilapia have shown that exogenous addition of sodium propionate alleviates intestinal barrier damage [47]. Therefore, it is reasonable to speculate that adding 150 mg/kg rutin to the high‐fava bean diets (60%) could promote intestinal health by increasing the abundance of propionate producing bacteria. *Rhodobacteraceae*, *Chloroplast*, and *Rothia* are all associated with the occurrence of infection and inflammation [48–50], the reduction in their abundance indicates that rutin may inhibit intestinal inflammation by suppressing the growth of pathogenic bacteria, but the specific mechanism needs further investigation. However, a high dose of rutin at 300 mg/kg has a poor improvement effect on the intestinal flora of crispy tilapia.

## 5. Conclusion

In summary, the inclusion of 150 and 300 mg/kg rutin in a 60% fava bean–based diet demonstrated no adverse effects on growth performance, while further improving muscle textural characteristics and enhancing nutrient deposition. Additionally, 150 and 300 mg/kg rutin significantly enhanced the AOC in muscle, liver, and intestine of crispy tilapia, effectively mitigating oxidative stress‐induced damage. This supplementation also alleviated liver injury through regulation of hepatic lipid metabolism. Notably, the 150 mg/kg rutin supplementation improved intestinal microbial composition, thereby substantially enhancing gut health in tilapia. In conclusion, rutin can effectively function as a nutritional supplement to alleviate liver and intestinal damage caused by 60% fava bean feed, while also improving muscle texture simultaneously. However, the setting of rutin concentration in this experiment has limitations, and the effect of rutin may change with the addition ratio of fava beans. Therefore, the most suitable addition amount of rutin in the fava bean–based diet still needs further exploration and verification.

## Disclosure

All authors have read and approved the manuscript.

## Conflicts of Interest

The authors declare no conflicts of interest.

## Author Contributions

Hua Wen conceptualized the study. Ming Jiang administered the project. Ke Cheng and Xinyao Zhang curated the data, investigated the study, and wrote the original draft. Lixue Dong, Di Peng, Yangyang Liu, and Juan Tian provided research methods. Dexing Zhu, Zhongbao Guo, Yongju Luo, and Apeng Liu were responsible for resources. Mingdian Liu and Ming Jiang acquisition of funds. Ke Cheng and Xinyao Zhang contributed equally to this work.

## Funding

The project was supported by the China Agriculture Research System (Grant CARS‐46), the Key Laboratory of Aquaculture genetic and breeding and Healthy Aquaculture of Guangxi Academy of Fishery Sciences (Grant GXKEYLA‐2023‐01‐12), and the Central Public Interest Scientific Institution Basal Research Fund (CAFS Number 2023TD09).

## Data Availability

The original contributions presented in the study are included in the article. Further inquiries can be directed to the corresponding author.
